# Clinical Validation of a Soft Wireless Continuous Blood Pressure Sensor During Surgery

**DOI:** 10.3389/fdgth.2021.696606

**Published:** 2021-07-22

**Authors:** En-Fan Chou, Shin Yu Celia Cheung, Hailey Christine Maxwell, Nicholas Pham, Michelle Khine, Joseph Rinehart

**Affiliations:** ^1^Department of Biomedical Engineering, University of California, Irvine, Irvine, CA, United States; ^2^Department of Medical Education, University of California, Irvine, Irvine, CA, United States; ^3^Department of Anesthesiology & Perioperative Care, University of California, Irvine Medical Center, Orange, CA, United States

**Keywords:** continuous non-invasive blood pressure monitoring, capacitive sensors, applanation tonometry, intraoperative, arterial pressure waveform

## Abstract

We test a new wireless soft capacitance sensor (CAP) based on applanation tonometry at the radial and dorsalis pedis arteries against the gold standard, invasive arterial line (A-Line), for continuous beat to beat blood pressure (BP) measurements in the Operating Room during surgical procedures under anesthesia in 17 subjects with the mean age and body mass index (BMI) of 57. 35 ± 18.72 years and 27.36 ± 4.20 kg/m^2^, respectively. We have identified several parameters to monitor in order to compare how well the CAP sensor tracks the entire hemodynamic waveform as compared to the A-Line. This includes waveform similarity, heart rate (HR), absolute systolic BP (SBP), diastolic BP (DBP), and temporal response to a vasopressor. Overall, the CAP sensor shows good correlations with A-Line with respect to hemodynamic shape (*r* > 0.89), HR (mean bias = 0.0006; SD = 0.17), absolute SBP, and DBP in a line of best fit (slope = 0.98 in SBP; 1.08 in DBP) and the mean bias derived from Bland-Altman method to be 1.92 (SD = 12.55) in SBP and 2.38 (SD = 12.19) in DBP across body habitus and age in OR patients under general anesthesia. While we do observe drifts in the system, we still obtain decent correlations with respect to the A-Line as evidenced by excellent linear fit and low mean bias across patients. When we post-process using a different calibration method to account for the drift, the mean bias and SD improve dramatically to −1.85 and 7.19 DBP as well as 1.43 and 7.43 SBP, respectively, indicating a promising potential for improvement when we integrate strategies to account for movement identified by our integrated accelerometer data.

## Introduction

Blood pressure (BP) is one of the core physiological measurements of interest in virtually all healthcare contexts as it provides insight into a patient's cardiac function, volume status, organ perfusion, and overall hemodynamic stability. It is typically monitored using a non-invasive sphygmomanometer, otherwise known as the BP cuff, and in higher-risk surgery may be monitored using an invasive arterial line. The arterial line (A-Line) is considered the gold standard in capturing beat-to-beat BP values to detect immediate fluctuations. This requires the insertion of a catheter into an artery, typically the radial or dorsalis pedis arteries. Because A-Lines are invasive, they are associated with an increased risk of complications including infection, thrombosis, and embolization (1–3). Clinicians may also experience difficulty cannulating the arteries so clinical expertise is required for proper insertion (4). As a result, 30% or less of patients in the Operating Room (OR) or Intensive Care Unit (ICU) receive A-Lines (3).

Instead, the overwhelming majority of patients even in hospital settings are only monitored intermittently using the BP cuff, which inherently lacks the temporal resolution to detect real-time fluctuations in hemodynamically labile patients. Moreover, such intermittent measurements have been observed to under or overestimate BP readings when compared with the A-Line (5, 6). In fact, a recent study has shown that BP cuff measurements are inaccurate (within ISO guidelines) up to almost 50% of the time (7).

Since blood pressure is a dynamic physiological parameter that changes constantly over time, non-invasive continuous blood pressure monitoring would reveal important hemodynamic information in real-time that is currently delayed by the intermittent BP cuff readings. This is especially important when labile BP warrants close monitoring–such as in the Emergency Room (ER), OR, or ICU settings. Furthermore, as healthcare moves towardz digital health, options for remote continuous BP monitoring (e.g., in ambulatory settings) would be incredibly useful in the management of hypertension, which affects roughly half of all American adults, as well as other medical conditions with vascular underpinnings (8). This is particularly important for personalized medicine—for example, to remotely monitor how patients respond to vasoactive pharmaceuticals.

For these reasons, non-invasive methods for continuous BP monitoring, or non-invasive blood pressure (NIBP), is an area of continued interest. Existing NIBP methods to capture continuous BP include optical techniques; derivations based on other vital sign measurements [such as pulse transit time (PTT)]; ultrasound technology; and tonometry. Concerning optical techniques, ClearSight (Edwards Lifesciences Corp., Irvine, CA) and CNAP (CNSystems, Graz, Australia) are both FDA-approved infrared photoplethysmography (PPG) devices that use the finger cuff volume clamp method. These devices are used preliminarily in the ICU or post-operative patients in the hospital because patients must remain stationary, the method is uncomfortable (a finger cuff repeatedly inflates), and they do not work well on patients with peripheral vascular disease or administration of high dose vasopressors (9–11). More recently, cuffless PPGs have been used to indirectly estimate BP using PTT, which is the time it takes for a BP waveform to propagate from one location to another. As such, estimates depend on physiological conditions and have been shown to be inaccurate in certain populations (12, 13). More recently, conformal ultrasound patches can monitor BP waveforms. However, they are yet to be wireless and require connection to a power source and a benchtop machine to display the data (14, 15). Finally, the last technology category, tonometry involves applying force over the artery to measure the pulsatile displacement of the vessel wall under applanation. Typically, the pressure transducers in this class have been rigid, bulky, and shown to be inaccurate in obese and cardiac patients (16, 17).

We have previously demonstrated the accuracy of a soft wearable capacitance sensor (CAP sensor) based on applanation tonometry for continuous non-invasive measurement of BP compared to the FDA-approved NIBP monitor Clearsight in a small cohort of healthy, young individuals (18). For clinical validation, however, it is important to compare against the gold standard in a clinical setting across body habitus and age. In this paper, we demonstrate calibration and comparison of pulse waveforms from the CAP sensor to A-Line measurements taken simultaneously in the intraoperative setting across 17 patients ranging in age from 24 to 79 years and importantly, with BMI from 24 (normal) to 34 (obese) kg/m^2^. Importantly, to do our comparison, we needed to develop an objective signal processing framework to analyze large data sets of different length scales with real-world noise and motion artifacts. Moreover, to make sense of the data, we needed to determine strategies to compare key parameters.

## Methods

BP data were acquired invasively and non-invasively using an A-Line and a CAP sensor, respectively. Surgical patients aged 18 to 99 years under general anesthesia in the OR setting who needed an A-Line placed as a standard of care were recruited at University of California Irvine Health between June 2020 and March 2021. Exclusion criteria were patients aged <18 years, refusal, or inability to give informed consent. All subjects gave informed consent for the study which was approved by the Institutional Review Board of the University of California (IRB no. 2019-5251).

### Measuring Devices and Systems

**Figure 1:**
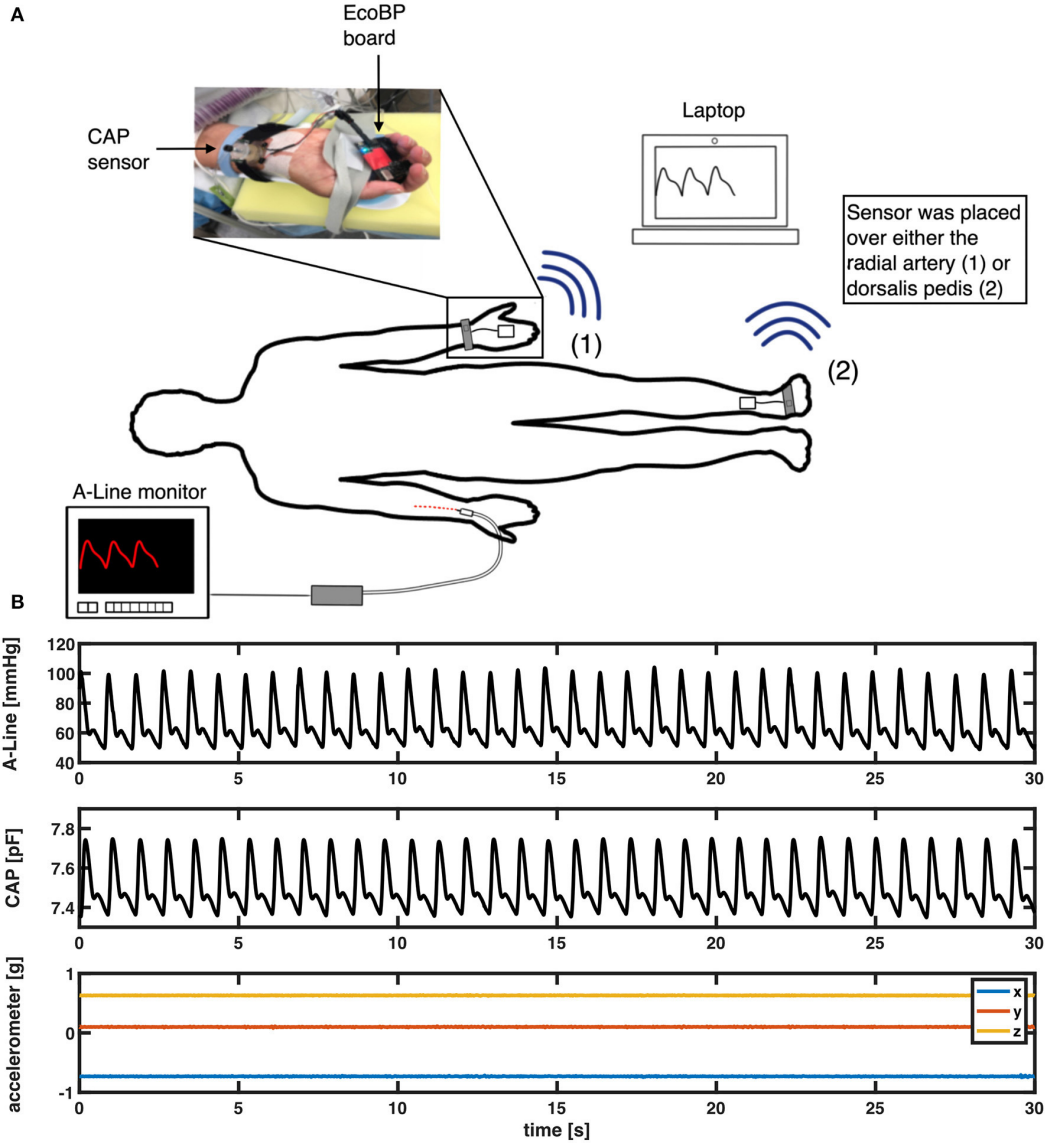
**(A)** Measurement setup in the OR. An intra-arterial line (A-Line) was inserted in the radial artery. The non-invasive system (CAP) was placed either on the radial artery or the dorsalis pedis artery depending on the procedure. **(B)** An example of a 30-s segment raw signal acquired from A-Line, the CAP sensor, and the accelerometer data that was used to compare waveform similarity, heart rate, and BP.

This study simultaneously employed two continuous BP measurement systems (Figure 1A). The A-Line was inserted into the radial artery and connected to a pressure transducer [ICU Medical Transpace^©^ IV Monitoring Kit (60″), REF no. 42584-05] and displayed on a monitoring system (GE Patient Data Module & Monitoring system, General Electric, Boston, MA). The A-Line signal was then captured at an average sampling rate of 100 Hz using a DAQ board (National Instruments cDAQ-9171 with NI 9234) with a custom application written in C#.

The non-invasive system comprises a soft capacitive pressure sensor (CAP) (18) and an EcoBP (19), an eco-friendly dual-channel custom data acquisition board that includes an inertial measurement unit (IMU). The CAP sensor was placed at the radial artery (or in two cases, the dorsalis pedis artery) for continuous arterial pressure measurement. The CAP signal was captured at a sampling rate of 90 Hz in single-channel mode and 45 Hz in dual-channel mode.

### Experimental Procedure

The A-Line was inserted into the radial artery on either arm and calibrated per hospital protocol. A BP cuff [CRITIKON(™) SOFT-CUF(™) REF SFT-A2-2A, GE Healthcare, Chicago, IL] was also placed on either arm for periodic measurements. The CAP sensor was placed over the radial artery, on the arm without the A-Line, and stabilized with slight pressure via mechanical fixation using velcro strap, sea-band (Sea-Band Ltd., Hinckley, Leicestershire, England), or Prelude Sync Radial Compression Device (Merit Medical Systems, Inc.). In cases where the radial artery was not readily available for placement, the dorsalis pedis artery was used (Figure 1). The EcoBP board was taped down onto the skin using Transpore tape (3M, Minnesota, USA).

After anesthesia induction, measurements were continuously collected from both CAP (non-invasive) and A-Line (invasive) systems by the anesthesiologist for a minimum of 15 min during the operation. The timing of administration of all intraoperative vasoactive medications was recorded in the electronic medical records (EMR).

### Data Extraction and Quality Assessment

**Figure 2:**
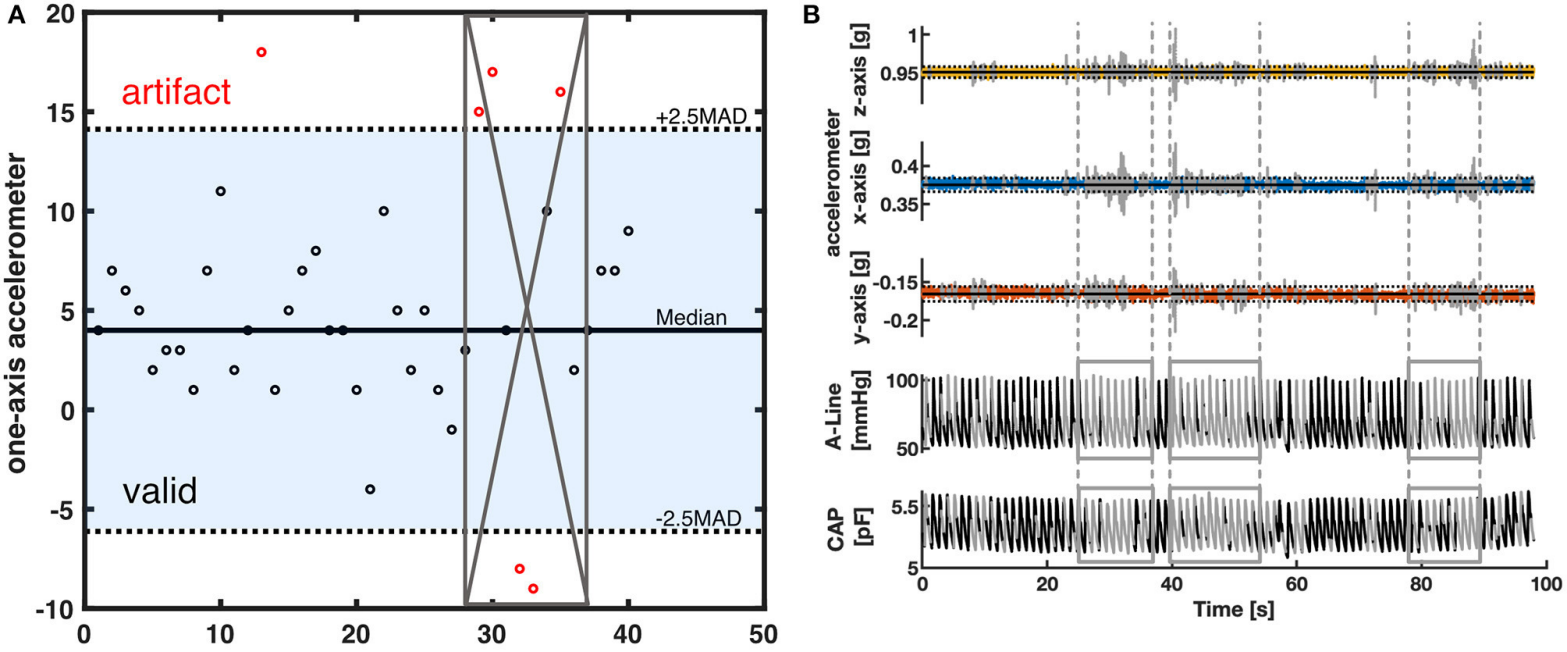
The artifact removal procedure using accelerometer data **(A)** Illustration of MAD filter and moving window. The horizontal solid line is the median value of the accelerometer dataset. The dotted lines are the 2.5-scaled MAD. Black circles represent the data within 2.5-scaled MAD. Red circles are considered outliers. The gray crossed area represents the invalid segment after a 50 percent threshold moving window with a window size of 8. **(B)** A representative segment showing how the data was filtered with a MAD filter and the moving window. The top three rows are the 3-axis of the accelerometer data. The horizontal solid line represents the median in each axis. The dotted lines are the 2.5-scaled MAD of each axis. The bottom two rows represent the corresponding A-Line and the CAP sensor data. The signal section colored gray and defined by the vertical lines was categorized as having excessive artifacts from the accelerometer data and thus removed.

The collected data was post-processed using Matlab (R2019b, The MathWorks, Natick, Massachusetts, USA). The raw signals were first spline interpolated to 500 Hz. To synchronize the signals recorded on different systems, cross-correlation was applied to the signal obtained by differentiating the discrete sequence of systolic peaks. After signal synchronization, clearly identified artifacts from invasive and non-invasive measurements were cleaned out. The A-Line data was visually screened by the authors for errors; excessively noisy sections defined by a sudden change of pressure >30 mm of mercury (mmHg) within two beats were removed. On the other hand, artifacts in the CAP signals were removed based on the movement data captured by the IMU. A 2.5-scaled median absolute deviation (MAD) filter was applied to accelerometer data on each of the 3 axes to detect outliers due to sudden and immoderate movement (Figure 2A). A 100-points (0.2 s) moving window was then applied and windows with at least 10% of data categorized as outliers were sliced out (Figure 2B). In addition to specific artifact removal techniques separately applied to A-Line and CAP signals, the intermittent BP cuff occlusions affected either A-Line or CAP measurements. Consequently, the affected data sections were cleaned out. It is worth noting that for consistency, any artifact-ridden data section in A-Line was also sliced out of CAP signal and vice-versa. After the removal of segments with excessive artifacts, the remaining signals were then considered as valid data for further analysis.

### Waveform Similarity Analysis

The diastolic and systolic pressure values and timestamps of each valid segment of data were identified for waveform analysis. A complete waveform was defined as the signal data bounded by two consecutive diastolic pressure values.

To analyze waveform shape similarity, corresponding A-Line and CAP sensor waveforms were normalized between 0 and 1. For each waveform, 0 was set as the average of the starting and ending diastolic pressure values, and 1 as the waveform maximum (systolic pressure value). A 0.1 threshold value was then applied to avoid false detection of minima (diastolic pressure).

### Heart Rate Monitoring

**Figure 3:**
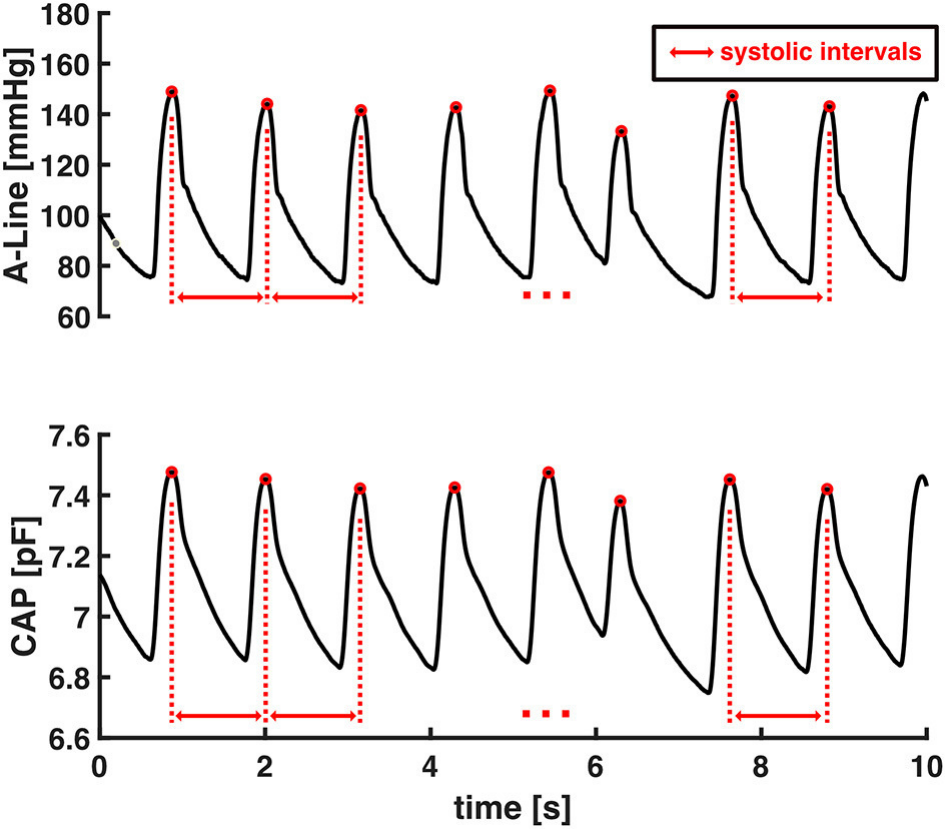
An example of the interval of two consecutive systolic pressures used to calculate HR.

HR is the reciprocal of the beat-to-beat time interval of the continuous blood pressure signal (Equation 1). The systolic intervals were obtained as the time interval from one systolic pressure value to the subsequent one as shown in Figure 3. The heart rates were calculated as the reciprocal of these systolic intervals.

The accuracy of HR estimation from capacitive pressure signal was evaluated by computing average HR values in each 30-s data window from valid segments of CAP sensor and then comparing against A-Line's. Each valid signal was sliced into 30-s non-overlapping windows starting from the beginning of the signal and any remaining data that was <30-s was excluded from HR computation.


(1) $$\begin{array}{r} Heart\;rate\;\left[bpm\right]=\frac{1}{beat-to-beat\;interval\;\left[min\right]} \end{array}$$

### Blood Pressure Comparison

Using valid data segments from A-Line and CAP sensor measurements, the CAP sensor's ability to accurately infer absolute BP is assessed.

Given that the CAP sensor's raw measurements are captured as capacitance values in picofarad (pF) and the A-Line's as absolute pressure in mmHg, a conversion from the CAP sensor's capacitance to absolute pressure in mmHg is needed to accurately assess the CAP sensor against A-Line. In this regard, we propose a calibration algorithm.

The initial step in the calibration process is the extraction of systolic and diastolic pressure signals from the raw A-Line and CAP sensor measurements. A 5-points median filter was applied to the extracted signals to smooth out detected false values. The filtered systolic and diastolic pressure signals were then split into individual 60-s segments for use in subsequent calibration steps. In a normal resting condition, a 60-s segment should contain 60–100 beats. The choice of 60-s as segment length is based on ClearSight's use of 70 beats between re-calibration (20).

**Figure 4:**
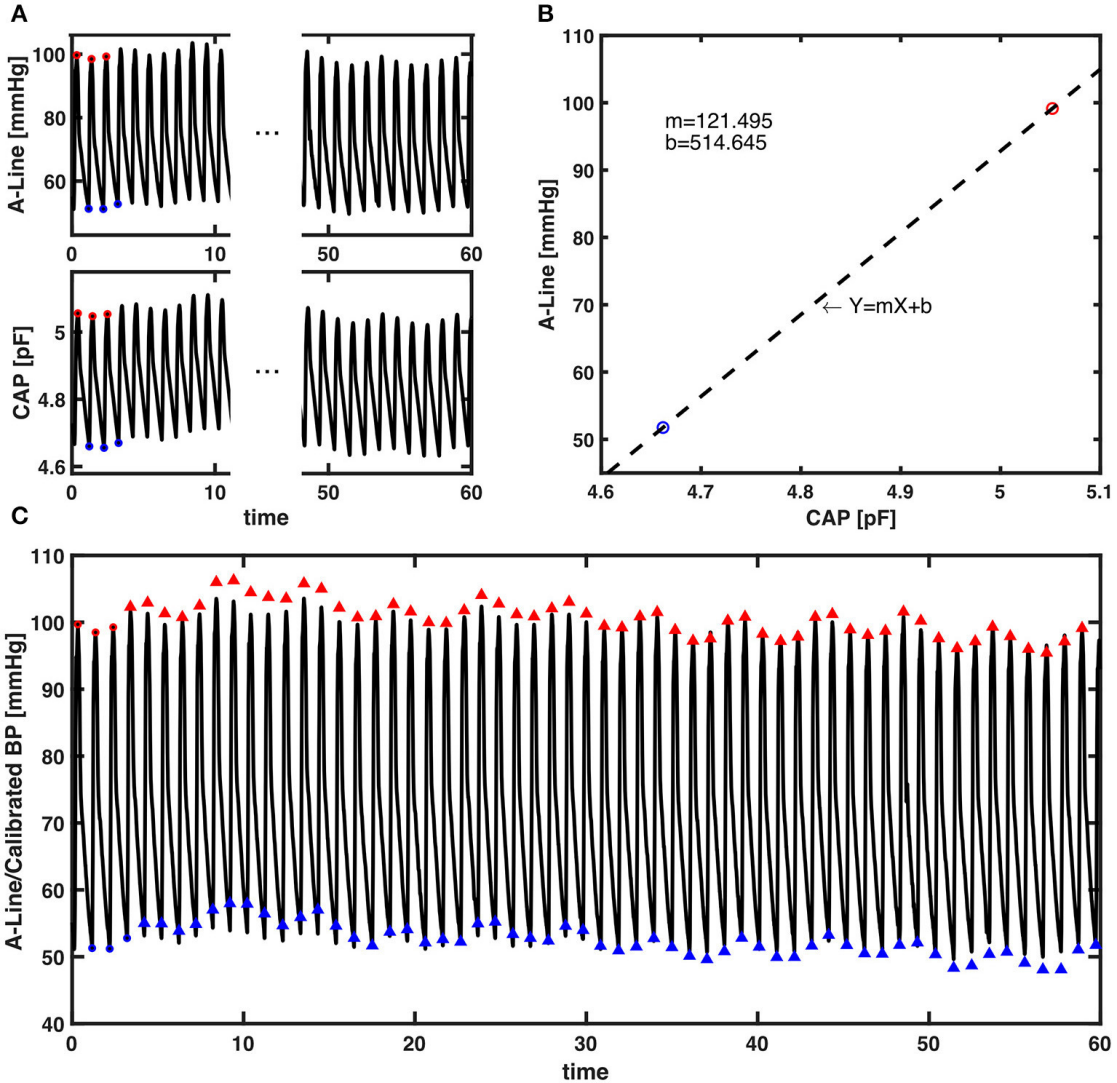
The presented calibration algorithm. **(A)** An example of a 60-s epoch raw signal acquired from A-Line and the CAP sensor. The red and blue circles are first three systolic and diastolic pressure values extracted for calibration from each signal, respectively. **(B)** Linear regression that models the relationship between the averaged systolic (red) and diastolic (blue) pressure values of A-Line and the CAP sensor from the first three beats. The dashed line is a linear regression line that forms the equation with m the slope and b the intercept. **(C)** The raw 60-s signal of A-Line, first three beats of SBP and DBP (red/blue circles), and the SBP and DBP values (red/blue triangles) calibrated from the CAP signal using the presented calibration algorithm.

The calibration algorithm took the average values of the first three beats in systolic and diastolic pressure every 60-s epoch. Sixty seconds was chosen for re-calibration periodicity as this is standard practice in ClearSight, a commercially available continuous NIBP device. A linear regression model was then created as the relationship between pressure measured in pF and mmHg. The remaining systolic and diastolic capacitance pressure values in the 60-s segment were then converted to the units of BP (mmHg). An example of the calibration algorithm can be found in Figure 4.

### Statistics

To assess the CAP sensor's ability in comparison to A-Line in an OR setting, several statistical methods were performed. Pearson correlation coefficient gives the linear correlation between features acquired from A-Line and the CAP sensor. Here, Pearson's *r* was computed to quantify the similarity between corresponding A-Line and CAP sensor normalized waveforms after aligning the two by their maxima (normalized systolic pressures) and how well the CAP sensor tracked BP compared to A-Line in vasoactive-drug-administered incidents. Moreover, the mean bias, SD, and 95% limits of agreement (estimated as the SD of the differences × 1.96) were calculated to understand the agreement between two methods by Bland-Altman method of paired measurements (21, 22). Mean HR in 30-s epochs and SBP and DBP in 60-s epochs derived from A-Line and CAP measurements were evaluated by this method. Lastly, the slope and *R*^2^-value of a simple linear fit were presented to evaluate the accuracy of beat-to-beat DBP and SBP using the proposed calibration algorithm.

## Results

We have identified several parameters to monitor in order to compare how well the CAP sensor tracks continuous BP as compared to the A-Line. This includes waveform similarity, HR, absolute SBP and DBP, and temporal response to a vasopressor.

### Participants

A total of 32 patients undergoing surgery requiring an A-Line placed were recruited during the study period, seven of whom were excluded due to the failure of data collection (data not recorded or data acquisition issues), five were excluded with obvious distortion in the measurements (artifacts or inaccurate sensor placement), and three others were excluded when the pulse signal from dorsalis pedis was affected by the intermittent pneumatic compression (IPC) device placed on the legs during the entire measurement. Thereafter, 17 patients (six male) with the mean age of 57.35 ± 18.72 years and mean body mass index (BMI) of 27.36 ± 4.20 kg/m^2^ were reported in the paper. Patients' demographics, the placement of CAP sensor, and procedure are shown in Table 1.

**Table 1:** Patients' demographic, CAP placement, and procedure.

**Subj. no**	**Age**	**Gender**	**BMI**	**Sensor placement**	**Procedure**
1	24	F	24.0	Radial	Open abdominal
2	49	M	26.6	Dorsalis pedis	Neck dissection
3	74	M	34.0	Radial	Microvascular decompression
4	78	F	33.5	Radial	Laparoscopic abdominal
5	23	F	20.0	Radial	Right calf sarcoma resection
6	55	F	32.0	Radial	Open abdominal
7	62	M	30.7	Radial	Radical, cystoprostatectomy
8	79	F	25.0	Radial	Open abdominal
9	61	F	27.0	Radial	Whipple
10	64	F	29.0	Radial	Whipple
11	22	F	30.2	Radial	Hip
12	72	M	26.6	Radial	Open abdominal
13	52	F	23.0	Radial	Ankle
14	59	F	28.0	Dorsalis pedis	Mandible tumor removal
15	61	F	21.6	Radial	Whipple
16	61	M	23.0	Radial	Open abdominal
17	79	M	31.0	Radial	Open abdominal

The post-processing method was used to remove obvious incorrect measurements and potential distortion affected by apparent artifacts. After removing for artifacts as heretofore described, the mean and standard deviation of the total amount of data included across the 17 studies was 46 ± 21%.

### Waveform Similarity

All the full-beat waveforms were included to understand the similarity of hemodynamic waveforms from two sites of the subject's body. A total of 20,090 full-beat waveforms were analyzed, which included non-invasive pulse waveforms from the radial artery and dorsalis pedis artery, to compare against BP derived from A-Line. Pearson's *r* is applied to indicate the strength of similarity between the two curves. Due to the length difference of valid segments in each dataset, the results are presented as averaged *r*-values with the quantity of full-beat waveforms as listed in Table 2. It is shown that the hemodynamic waveforms acquired from two different locations, regardless of the invasiveness of the acquisition technique, of the same patient have a very strong linear relationship (23). Besides, the two studies with averaged *r* lower than 0.9 (Subject 2 and Subject 14) were the only two studies having the pressure sensor placed on dorsalis pedis artery rather than the radial artery.

**Table 2:** Mean and standard deviation of Pearson correlation coefficient *r* across 17 subjects.

**Subj. no**	***N***	**Mean**	**STD**
1	343	0.9920	0.0041
2	281	0.8888	0.0339
3	593	0.9410	0.0238
4	861	0.9046	0.0198
5	537	0.9459	0.0315
6	533	0.9332	0.0948
7	1097	0.9739	0.0262
8	664	0.9362	0.0296
9	466	0.9310	0.0196
10	834	0.9818	0.0113
11	442	0.9732	0.0087
12	513	0.9854	0.0261
13	3,063	0.9807	0.0126
14	1,470	0.8983	0.0308
15	3,742	0.9473	0.0192
16	2,699	0.9277	0.0571
17	1,952	0.9839	0.0041

*Values of each subject were calculated according to the number of valid waveforms (N). STD, standard deviation*.

### Heart Rate Monitoring

**Figure 5:**
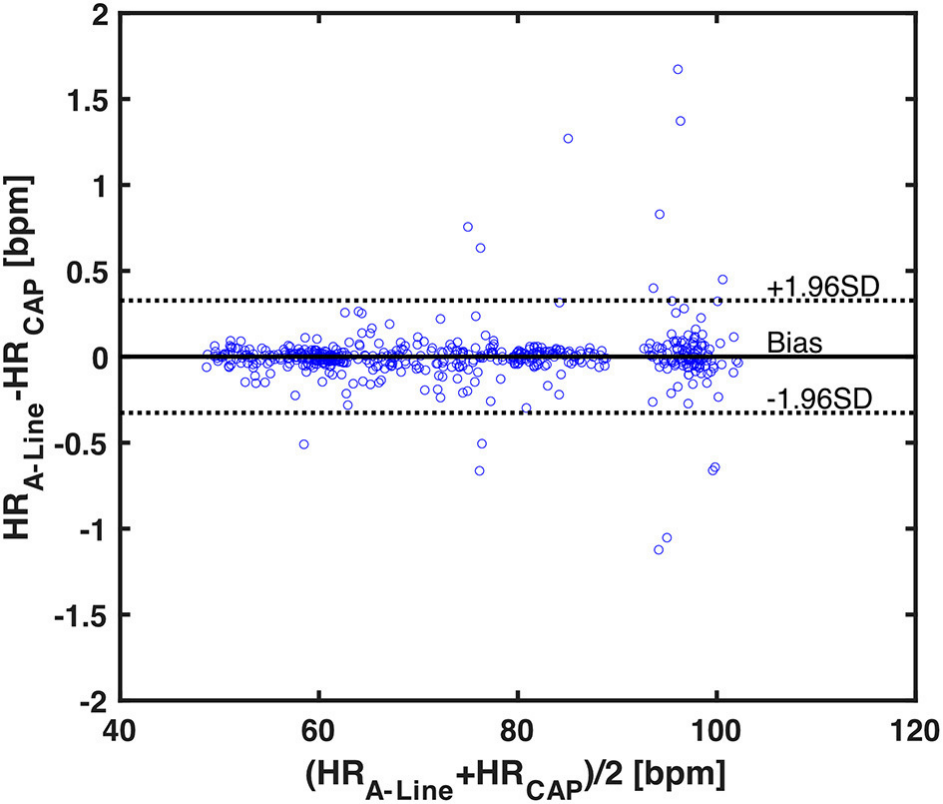
A Bland-Altman plot of 562 averaged heart rates calculated from 30-s valid segments across 17 patients. Each blue circle is one averaged HR data. The black horizontal solid and dotted lines represent the mean bias and the upper and lower 95% limits of agreement, respectively. The mean bias in differences is 0.0006, upper 95% limit is 0.3272, and lower 95% limit is −0.3259.

To assess the accuracy of heart rate detection across all the patients in the study, a total of 562 30-s valid segments for both A-Line and CAP were extracted and compared in the mean and standard deviation of heart rate. The mean bias and SD of mean HR in two measurements are 0.0006 and 0.1666 bpm, respectively. The 95% limits of agreement lie in the range of −0.3259 and 0.3272 bpm (Figure 5).

### Blood Pressure Comparison

**Figure 6:**
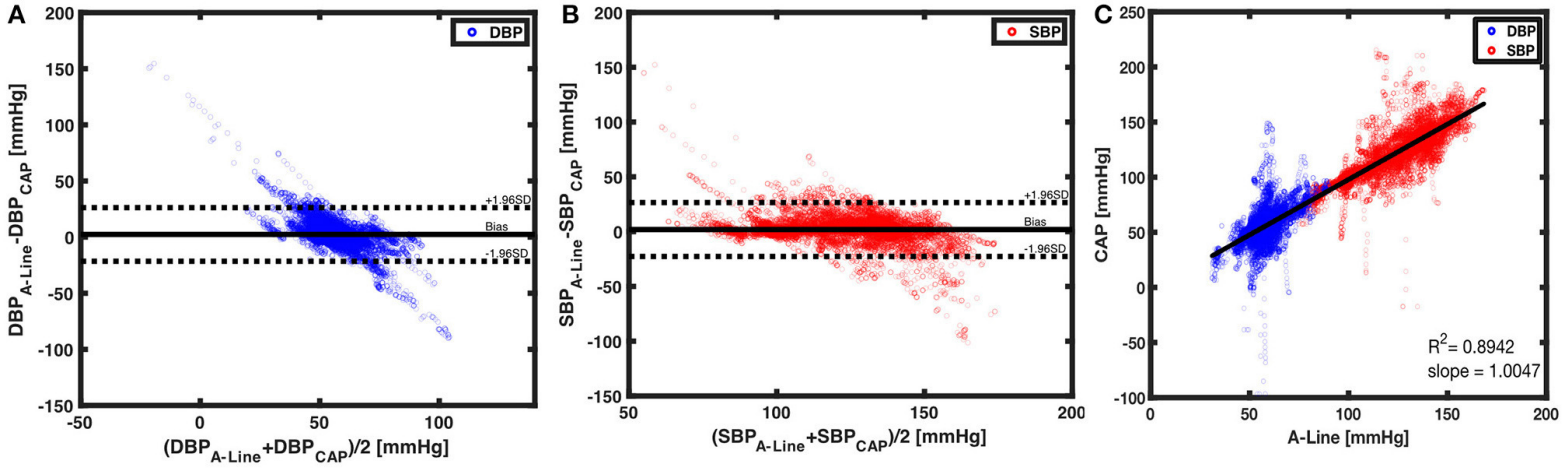
Beat to beat BP comparison using Bland-Altman method and linear regression. **(A)** With 14,645 paired data points of DBP (blue circles), a mean bias of 2.3842 (solid black line) and an SD of 12.1908 were calculated. The dotted black lines are the upper (26.2782) and lower (−21.5098) 95% limits of agreement. On the other hand, **(B)** 14,674 paired measurements of SBP (red circles) were compared. The mean bias of 1.9153 (horizontal black solid line) and an SD of 12.5525 were calculated. The 95% limits of agreement ranged from −22.6876 to 26.582. **(C)** The CAP sensor was compared against the A-Line using a linear regression model over 29,319 data points from valid 60-s segments. A linear fit slope of 1.0047 shows the two measurements in good correlation.

A total of 29,319 BP values (14,645 diastolic and 14,674 systolic) were obtained and compared from 209 60-s segments. The mean bias of overall DBP and SBP are 2.3842 (SD = 12.1908) and 1.9153 (SD = 12.5525), respectively. The limits of agreement in DBP are from −21.5098 to 26.2782 mmHg and for SBP from −22.6876 to 26.5182 mmHg (Figures 6A,B). To understand the correlation of BP measurements within two systems, a best-fit line with a slope of 1.0047 was derived (Figure 6C). The separated plots of SBP and DBP measured by A-Line and the CAP sensor can be seen in Supplementary Figure 2.

**Figure 7:**
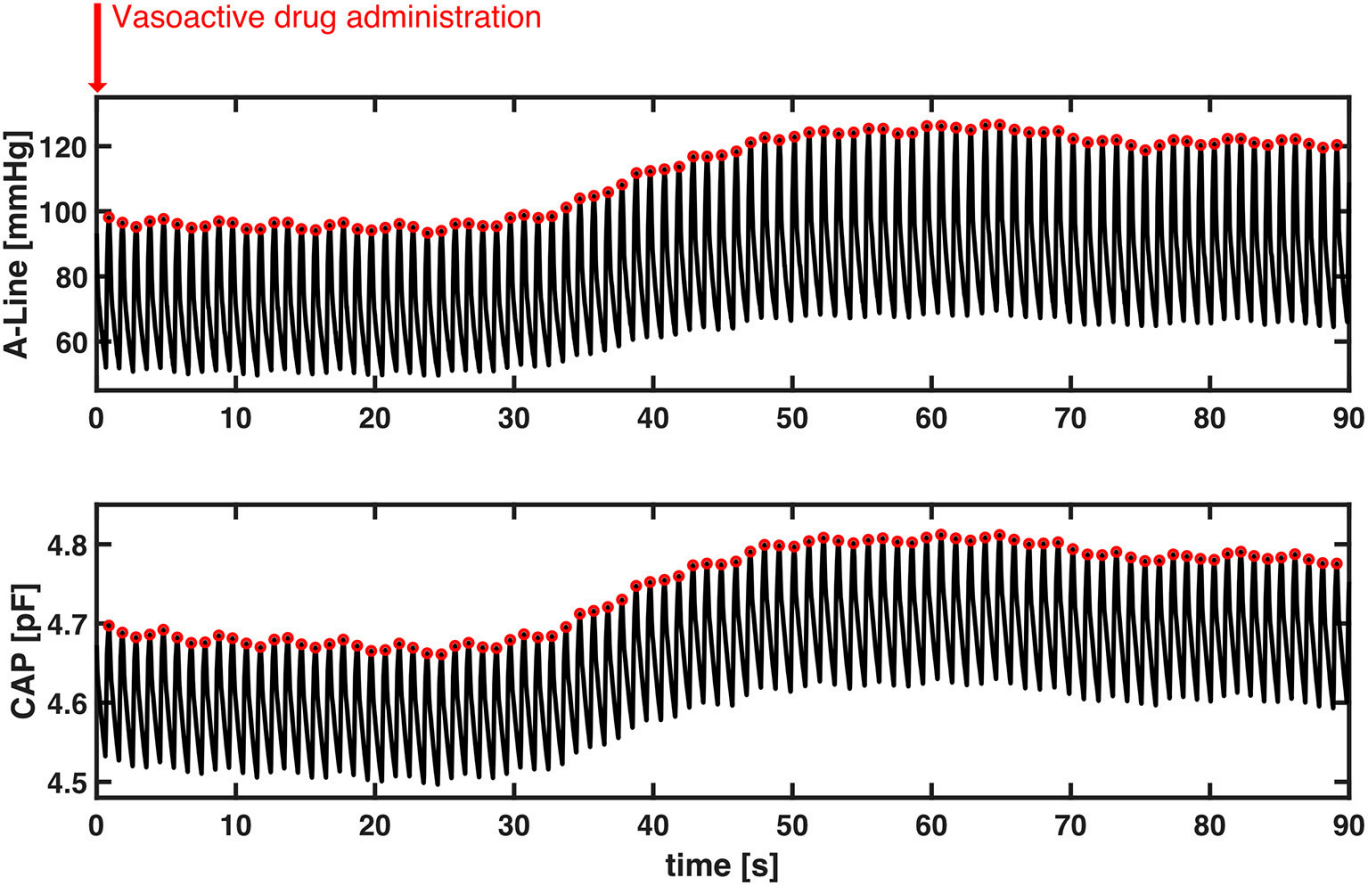
A representative section of the temporal response to vasoactive drug administration in both A-Line and CAP signals. Without motion artifacts, BP increased about 30 s simultaneously after Ephedrine and Vasopressin were administered. Systolic peak values of both A-Line and CAP signals were detected as the red circles. The Pearson correlation is 0.9973.

Lastly, we observed the BP change after the vasoactive drugs were administered. A total of 20 events were identified according to physician observation and the EMR across nine patients (no vasoactive drug administration was observed while the other eight patients were recorded). Three events were excluded due to movement artifacts caused by either the surgeon or the periodic BP cuff measurement. The mean duration of the 17 events was 55.4 (SD = 29.8) seconds. The Pearson's *r* of SBP between A-Line and CAP was 0.82 ± 0.28 (mean±SD). Figure 7 shows a representative event in which both BP measured from A-Line and the CAP sensor increased 30 s after Ephedrine and Vasopressin were administered. This is unlike sensors based on PPG which have difficulty tracking fast changes in BP (24).

## Discussion

The major finding of the study is that the CAP sensor can non-invasively track BP across different body types and ages in an OR setting under general anesthesia. In particular, the study showed the sensor's capability of capturing hemodynamic waveforms from different arterial sites with high fidelity compared to the A-Line. Additionally, we found a good correlation in HR monitoring. Concerning beat-to-beat BP monitoring, we showed a low mean bias of both SBP and DBP.

### Clinical Application

At present, we have demonstrated an ability to accurately track blood pressure with high confidence. While the present performance may be improved over time and is not a replacement for continuous arterial monitoring, there would nevertheless be clinical utility even with the present sensor as an adjunct to non-invasive monitoring (which is used universally during surgery). The NIBP monitoring could be used as it currently is, and the CAP sensor recalibrated with each cycling of the non-invasive cuff. Meanwhile, the CAP sensor would provide continuous monitoring in the periods between cuff cycles, catching hypotension faster and allowing for more rapid treatment, as well as allowing for more rapid assessment of other interventions like narcotics or anti-hypertensives.

### Subject Inclusion

Fifteen out of 32 datasets had been excluded from the study for the following reasons: failure of data collection, obvious distortion (in the A-line data or from obvious misplacement of the CAP), and the simultaneous use of the IPC device. Improved instructions on applying the CAP sensor and/or improved applanation (strap) design should improve the ability to capture more datasets in the future.

### Single-Channel CAP Sensor

As previously mentioned, the 17 studies had been done either using a single or dual-channel CAP sensor. For the dual-channel CAP sensor, the second channel may be useful as a neighbor reference of the main arterial pressure signal. However, we have not yet investigated the benefits of dual-channel acquisitions and as a result, only one capacitance reading is included for all the data analysis in the study. We believe a multi-channel CAP sensor will greatly improve blood pressure monitoring by eliminating the need for frequent recalibrations.

### Artifact Detection and Quality Assessment

There are a number of confounding factors inherent in any continuous physiological monitoring. This study was done in an OR setting to investigate the validity of the CAP sensor for a specific reason: we can account for and eliminate many of these variables. For instance, we have the BMI of every patient and whether they have hypertension via EMR. We found no correlation between BMI or hypertension and signal accuracy. The other major confounding factor is movement artifact.

From the development of the CAP sensor, it was found to have a low tolerance to movements. Compared to the previous study published using the CAP sensor in a relatively controlled subject group, this study was done in a real clinical setting where the measurement could be affected by multiple challenges.

One obvious difference of BP measurement with non-invasive technology is that the data is obtained outside the subject's body. With different body sizes and ages of a patient, the tissue layer between the CAP sensor and the artery may create artifacts that directly influence the pressure measurement. For instance, the mechanical fixation on an elder patient with loose skin can be more challenging. Besides, the tightness and positioning of the wristband affected the signal quality. This also explains the reason why the study included multiple mechanical fixation ways.

Furthermore, human-caused artifacts such as the surgeon's need to interact with the patient's body or the interference from electronic noises were inevitable in the OR. We chose to remove the artifacts of the A-Line signal by eye considering limited information. A similar data exclusion method has been previously used by several other groups (16, 25, 26). In this case, it can be improved by including video recordings to have a better understanding of the artifact sources. On the other hand, the accelerometer data from the EcoBP was used to filter significant motion artifacts. The MAD filter is a robust measure of statistical dispersion. Although a 2-scaled MAD or 3-scaled MAD may serve a similar purpose of artifact removal, a 2.5-scaled MAD was chosen as an adequate threshold to separate the significant motion artifacts in the study while conserving a reasonable percentage of valid data.

We also observed baseline drift in some datasets from the CAP signal. In most cases, a downward drift occurred linearly or exponentially. The cause might be due to the movement or unknown factors. We expect a future study to resolve the issue. Despite the aforementioned challenges, it is clear that after the objective signal processing strategy outlined above, the CAP sensor showed a good correlation with A-Line during surgery and can provide a wealth of information about the hemodynamic waveform. In addition to the excellent slope value, the average mean bias and standard deviation across 15 patients were low.

### Waveform Similarity

Pearson correlation was used to evaluate the degree of the linear relationship between A-Line and CAP waveforms. Similar work had been previously done (27).

**Figure 8:**
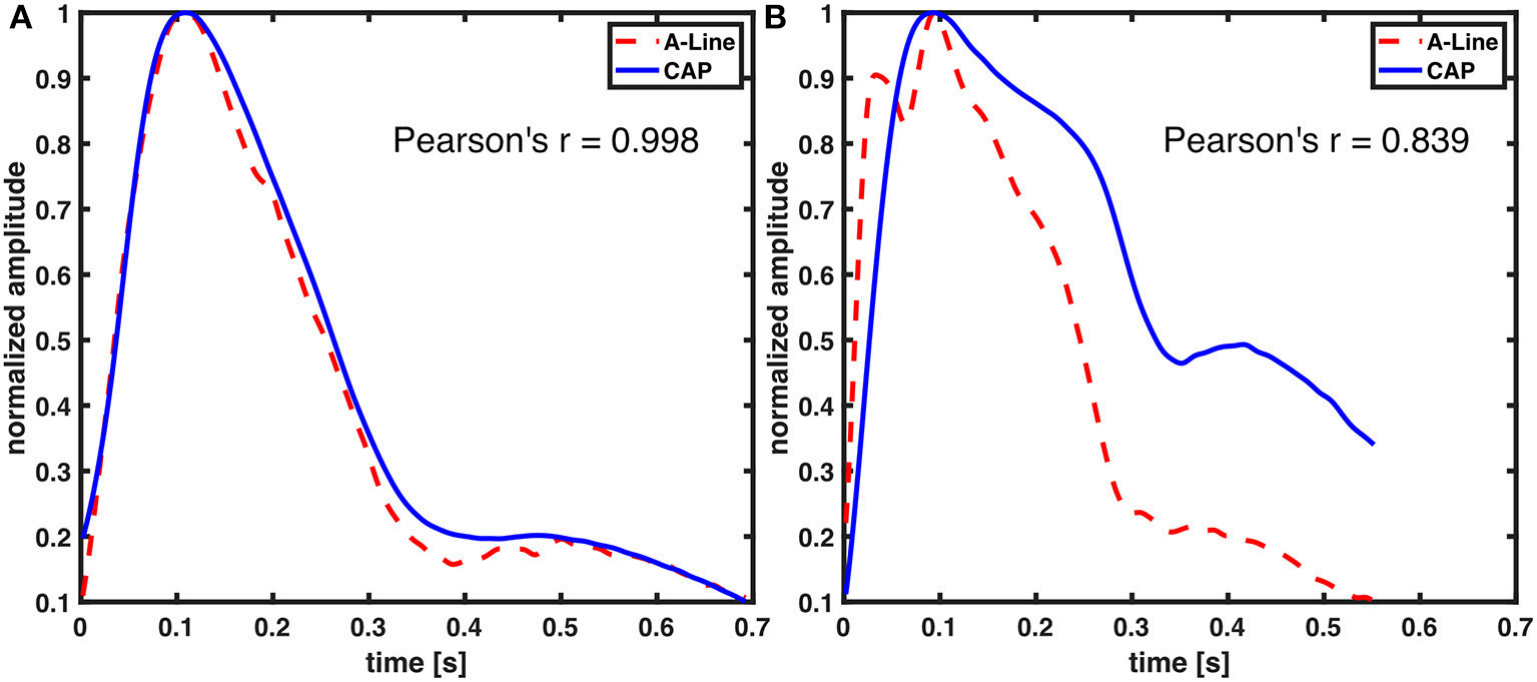
Comparison of A-Line and CAP waveform similarity. Both of the waveforms were derived from radial arteries. Representative figures with **(A)** high and **(B)** low Pearson correlation coefficient.

In this study, the focus for waveform similarity analysis was to compare full-beat wave shape. Consequently, the waveforms were normalized and aligned by the systolic pressure to avoid the time delay due to measuring from different sites of the body. A very strong correlation between A-Line and CAP sensor measurements was reported. Although not all waveforms from both signals perfectly match visually (Figure 8), it is in fact a characteristic of the peripheral arterial pressure waveform measured across different arterial sites. This waveform distortion effect is due to the individual physical characteristics of the arterial tree (28, 29). Additionally, the applanation tonometry waveforms were detected indirectly as the pulse signal propagated through the artery wall and the tissues. The CAP sensor not only can capture the components of the arterial pressure waveform (diastolic pressure, systolic pressure, upstroke of systole, dicrotic notch, etc.) but also potentially shows differences in the pulse pressure profile in the radial artery (upper extremity) and the dorsalis pedis (lower extremity) as shown in Supplementary Figure 1. This implies that the CAP sensor could acquire similar hemodynamic waveforms to the A-Line but non-invasively in an OR setting.

### Limitations

The current study is subject to several limitations. Because this is a retrospective study, we went back to calibrate to the A-Line, after completion of data collection. Going forward, a sensor that calibrates to the BP cuff in real-time would be advantageous. The greatest challenge that we had was accounting for motion artifacts and drifts in the signal after motion. We, therefore, had to splice out many sections of the data to account for active movement as captured by the accelerometer. However, outside the window that we spliced out (during active movement), we observed the movement caused a longer-term drift over time. We did not account for such drifts in our analysis but had we done so, the results would be even better. This is evidenced by the fact that if we instead calibrated to the first, middle, and last point of each 60-s epoch (instead of just the average of the first three beats), the averaged mean bias and average SD go down considerably, from 2.3842 ± 12.1908 to −1.8477 ± 7.1906 in DBP and from 1.9153 ± 12.5525 to 1.4324 ± 7.4321 in SBP (Supplementary Figure 3). This indicates that the CAP signal drifts over the 60-s epoch. In future iterations, we would like to not toss away all the sections with movement, but use machine learning along with the parallel sensor channels to correct for the motion (30). We would also like to model out the drifts caused by this motion so we can objectively account for and subtract it from the signal. This would undoubtedly improve our agreements with the A-Line.

Overall, in summary, we show that the CAP sensor is a promising technology that has good agreement with the A-Line regarding the hemodynamic shape, heart rate, SBP, and DBP across body habitus and age in OR patients under general anesthesia. While we do observe drifts in the system, we still obtain good correlations with respect to the A-Line as evidenced by excellent linear fit and averaged mean bias and standard deviation across all patients. Moreover, CAP seems to be able to track fast changes in blood pressure well, which is critical to monitoring hemodynamically unstable patients.

## Data Availability Statement

The raw data supporting the conclusions of this article will be made available by the authors, without undue reservation.

## Ethics Statement

The studies involving human participants were reviewed and approved by University of California Irvine Institutional Review Board. The patients/participants provided their written informed consent to participate in this study.

## Author Contributions

JR: study design, lead investigator of IRB, data collection and overseeing all data collection and signal processing, and paper writing. HM and NP: data collection. E-FC and SC: data analysis and writing. MK: data analysis, writing, and providing sensors. All authors contributed to the article and approved the submitted version.

## Conflict of Interest

MK has financial interests in Vena Vitals as the scientific co-founder of the company and as such, owns equity in Vena Vitals, which is in addition to her salary from the University of California, Irvine. The nature of this financial interest and the design of the study have been reviewed by the UCI Conflict of Interest Oversight Committee (COIOC). The COIOC has determined that the researcher's financial interests are appropriately managed to avoid compromising the quality or reliability of the study and furthermore, the UCI Institutional Review Board has determined that appropriate safeguards are in place to avoid adversely affecting your safety and welfare. The remaining authors declare that the research was conducted in the absence of any commercial or financial relationships that could be construed as a potential conflict of interest.

## Supplementary Material

Supplementary Figure 1 Hemodynamic waveform captured from **(A)** radial artery and **(B)** dorsalis pedis. The black dotted line represents the mean arterial pressure of each wave.

Supplementary Figure 2 BP measured by A-Line and the CAP sensor from valid 60-s segments using the proposed calibration method. **(A)** A linear fit slope of 1.0785 and *R*^2^ of 0.329 were derived from 14,645 DBP in blue circles. The black solid line across the blue circle is the best linear fit line. **(B)** A linear fit slope of 0.9774 and *R*^2^ of 0.685 were derived from 14,674 SBP in red circles. The black solid line across the red circle is the best linear fit line.

Supplementary Figure 3 Bland-Altman plot using **(A)** 14,645 diastolic and **(B)** 14,674 systolic BP showing level of agreement from valid 60-s segments obtained by A-Line and the CAP sensor. The horizontal black solid, dashed, and dotted lines represent the mean bias, limits of agreement, and the zero line, respectively. The red error bars on the black dashed limits of agreement lines are the 95% confidence intervals of the upper and lower limits.
