# Supplementary figures and images for: Clinical Validation of a Soft Wireless Continuous Blood Pressure Sensor During Surgery
Source: Front Digit Health. 2021 Jul 22;3:696606. doi: 10.3389/fdgth.2021.696606 (PMC8521971; doi:10.3389/fdgth.2021.696606)

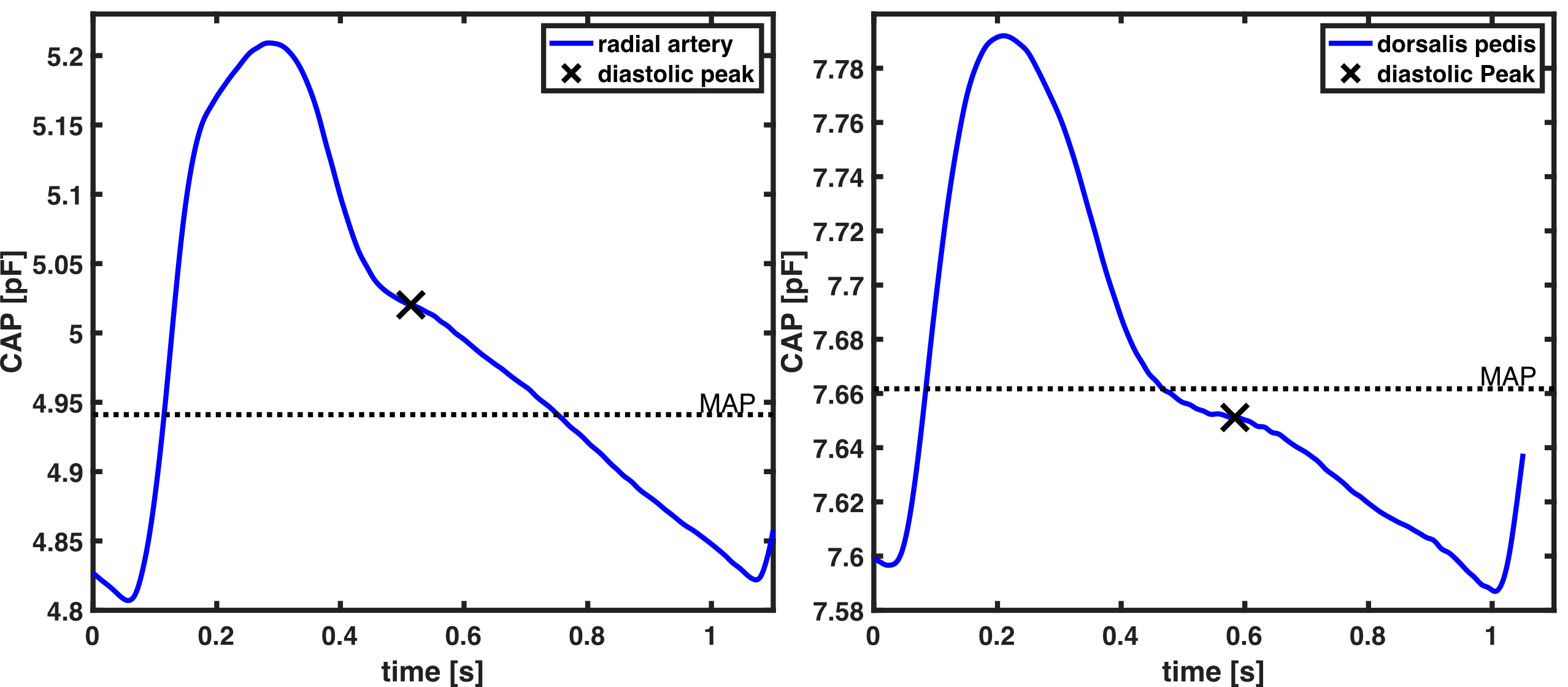

Supplement: Supplementary Figure 1 — Hemodynamic waveform captured from (A) radial artery and (B) dorsalis pedis. The black dotted line represents the mean arterial pressure of each wave. [file Image_1.JPEG]

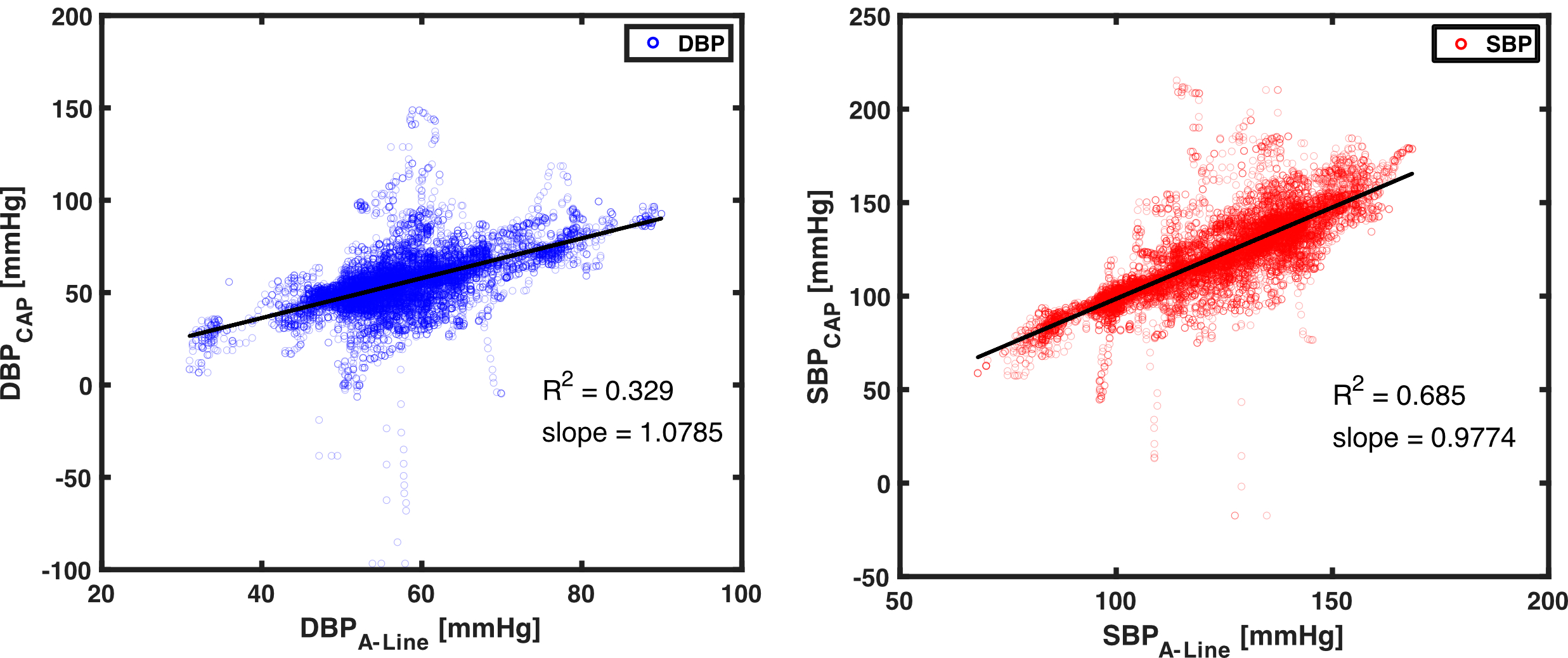

Supplement: Supplementary Figure 2 — BP measured by A-Line and the CAP sensor from valid 60-s segments using the proposed calibration method. (A) A linear fit slope of 1.0785 and R2 of 0.329 were derived from 14,645 DBP in blue circles. The black solid line across the blue circle is the best linear fit line. (B) A linear fit slope of 0.9774 and R2 of 0.685 were derived from 14,674 SBP in red circles. The black solid line across the red circle is the best linear fit line. [file Image_2.JPEG]

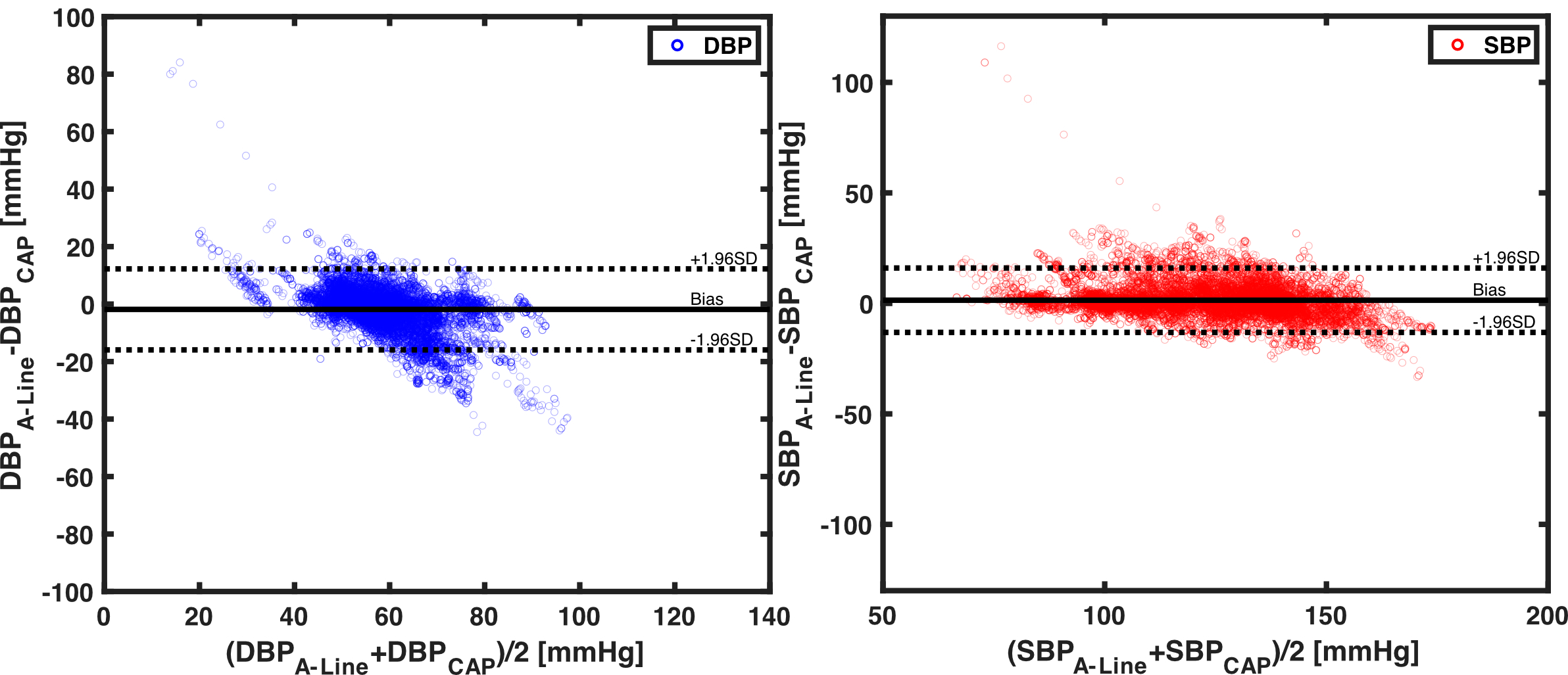

Supplement: Supplementary Figure 3 — Bland-Altman plot using (A) 14,645 diastolic and (B) 14,674 systolic BP showing level of agreement from valid 60-s segments obtained by A-Line and the CAP sensor. The horizontal black solid, dashed, and dotted lines represent the mean bias, limits of agreement, and the zero line, respectively. The red error bars on the black dashed limits of agreement lines are the 95% confidence intervals of the upper and lower limits. [file Image_3.JPEG]
